# Identification of two distinct structural regions in a human porcine endogenous retrovirus receptor, HuPAR2, contributing to function for viral entry

**DOI:** 10.1186/1742-4690-6-3

**Published:** 2009-01-14

**Authors:** Katherine T Marcucci, Takele Argaw, Carolyn A Wilson, Daniel R Salomon

**Affiliations:** 1 Department of Molecular and Experimental Medicine, The Scripps Research Institute, La Jolla, CA, 92037, USA; 2 Division of Cellular and Gene Therapies, Center for Biologics Evaluation and Research, U.S. Food and Drug Administration, Bethesda, MD, 20892, USA; 3 Department of Pediatrics, Children's Hospital of Philadelphia, Philadelphia, Pennsylvania, 19104, USA

## Abstract

**Background:**

Of the three subclasses of Porcine Endogenous Retrovirus (PERV), PERV-A is able to infect human cells via one of two receptors, HuPAR1 or HuPAR2. Characterizing the structure-function relationships of the two HuPAR receptors in PERV-A binding and entry is important in understanding receptor-mediated gammaretroviral entry and contributes to evaluating the risk of zoonosis in xenotransplantation.

**Results:**

Chimeras of the non-permissive murine PAR and the permissive HuPAR2, which scanned the entire molecule, revealed that the first 135 amino acids of HuPAR2 are critical for PERV-A entry. Within this critical region, eighteen single residue differences exist. Site-directed mutagenesis used to map single residues confirmed the previously identified L109 as a binding and infectivity determinant. In addition, we identified seven residues contributing to the efficiency of PERV-A entry without affecting envelope binding, located in multiple predicted structural motifs (intracellular, extracellular and transmembrane). We also show that expression of HuPAR2 in a non-permissive cell line results in an average 11-fold higher infectivity titer for PERV-A compared to equal expression of HuPAR1, although PERV-A envelope binding is similar. Chimeras between HuPAR-1 and -2 revealed that the region spanning amino acids 152–285 is responsible for the increase of HuPAR2. Fine mapping of this region revealed that the increased receptor function required the full sequence rather than one or more specific residues.

**Conclusion:**

HuPAR2 has two distinct structural regions. In one region, a single residue determines binding; however, in both regions, multiple residues influence receptor function for PERV-A entry.

## Background

Pigs are considered as suitable alternatives for human cell, tissue and organ sources due to physiological and size compatibilities and development of pathogen-free herds. However, one concern with the use of pigs in clinical xenotransplantation is Porcine Endogenous Retrovirus (PERV), a potential zoonotic gammaretroviral infection risk. While productive PERV infection in patients exposed to porcine cells or tissues after xenotransplantation has not been documented [1-13], the fact is that there is little evidence of long-term survival of pig tissues in a human host. Thus, it is still important to understand the molecular determinants of human-tropic receptor-mediated PERV infection as interest in commercialization of pig donor xenotransplantation continues to evolve with at least one biotechnology company doing clinical trials with pig islet transplants.

PERV-A [14-16] and PERV-B [15-17] are human-tropic viral species while PERV-C [18,19] is not. PERV-A enters human cells via one of two receptors, HuPAR1 or HuPAR2 [20], while the human receptor for PERV-B remains unknown. Even so, PERV-A represents the most significant risk for human infection since it is present in the pig genome at levels higher than PERV-B [15] and can recombine with PERV-C to produce higher titer human-tropic PERV-A/C recombinants [21]. Therefore, understanding the receptor determinants that contribute to PERV-A and PERV-A/C entry is a logical step in the science-based risk assessment of possible PERV transmission and infection in clinical xenotransplantation.

Gammaretroviral entry requires viral envelope binding to a multiple transmembrane domain cell-surface receptor and subsequent viral and plasma membrane fusion. Most gammaretroviruses use one cell-surface molecule for entry. E-MLV uses mCAT1 [22]; FeLV-T [23], GALV [24] use Pit1; A-MLV [25,26] uses Pit2; RD114 uses ASCT2 [27,28]; X-MLV and P-MLV [29] use the X-receptor; FeLV-C [30,31] uses FLVCR1; and FeLV-A [32] uses THTR1. Feline Leukemia Virus T (FeLV-T) is the exception in that it also requires a soluble cofactor, FeLV infectivity X-essory protein (FeLIX) [23], in addition to its primary cell-surface receptor, Pit1 [33]. Chimeras of permissive and non-permissive orthologs have identified receptor regions required for entry for all the receptors described above but THTR1. Extracellular loop(s) are important for the viral receptor function of mCAT1 [34,35], Pit2 [36,37], X-receptor [38], ASCT1, ASCT2 [39] and FLVCR1 [30], while both a transmembrane [40] and an intracellular [41-43] region are required for Pit1. In addition, BaEV [27,28] and HERV-W [44] can use either ASCT1 or ASCT2, while 10A1 MLV [42] can use either Pit1 or Pit2. However, functional mapping between the individual receptors in such homologous pairs has not been done.

PERV-A can use either HuPAR1 or HuPAR2 to enter human cells or non-permissive cell lines expressing the receptors (e.g. SIRC and NIH3T3) [20]. Structurally, the 445 amino acid HuPAR1 protein and 448 amino acid HuPAR2 protein share 86.5% sequence identity. Current experimental evidence [45] and topology prediction algorithms [46,47] support an eleven transmembrane model with an intracellular N-terminus and an extracellular C-terminus. In contrast, the N- and C-termini of all the other known gammaretroviral receptors are either both intracellular or both extracellular. While most gammaretroviral receptors are small metabolite transporters (reviewed in [48] and [49]), HuPAR1 was recently identified as a G-protein coupled receptor for gamma-hydroxybutyrate (GHB) in the brain [50], although lack of the canonical 7 transmembrane domains characteristic of G-protein-coupled receptors, inadequate controls in the reported data, and absence of independent verification, leaves the major conclusion open to further interpretation. The endogenous function of HuPAR2 is unknown and the function of HuPAR1 in other tissues has not been tested.

The structure-function determinants of PERV-A entry have not been extensively studied for HuPAR1 and HuPAR2. Presently, leucine 109 (L109) in the second predicted extracellular loop, is the only residue that has been shown to be essential for HuPAR2 function by mediating PERV-A binding. In the non-functional HuPAR orthologs of *Mus musculus *and *Mus dunni*, this residue is a proline and explains the resistance of the murine species [45]. Additionally, the initial receptor characterizations indicated that HuPAR2 was approximately ten-fold more functional than HuPAR1 for PERV-A infection [20]. The structural basis for this functional difference is unknown.

In this manuscript we confirm the role of L109 in viral envelope binding and identify seven new residues in the N-terminal 135 amino acids that each influence HuPAR2 function significantly for PERV-A entry but without affecting PERV envelope binding.

Using chimeras constructed between HuPAR1 and HuPAR2, we demonstrate that a second region comprised of the third extracellular loop, the sixth transmembrane domain, the third intracellular loop and the seventh transmembrane domain (a.a. 152–285) of HuPAR2 is responsible for the ten-fold functional superiority of HuPAR2. We have identified two regions in this gamma retroviral receptor with distinct structure-function relationships that either determine or enhance HuPAR2 function in human-tropic PERV infection.

## Methods

### Cell lines: maintenance, transfection and selection

293 T cells were maintained in DMEM (Gibco) supplemented with 10% fetal bovine serum (HyClone), 5% 1 M Hepes (Gibco), 5% 100 mM sodium pyruvate (Gibco) and 5% 100× penicillin-streptomycin-glutamine (Gibco). SIRC cells (rabbit cornea, ATCC CCL-60) were maintained in MEM + L-glutamine (Gibco) supplemented with 10% bovine serum (HyClone, Logan, UT), 5% 1 M Hepes (Gibco), and 5% 100× penicillin-streptomycin. SIRC cells were transfected with 3 μg plasmid encoding the PAR cDNA by nucleofection (Amaxa). Stable cell lines were selected with 400 μg/mL Zeocin (Invitrogen). After 3–4 weeks, cell lines were sorted for eGFP selection. Sorted cell lines were maintained without antibiotics and remained stable.

### Constructs

Starting with a molecular clone, PERV-A14/220 (GenBank AY570980) [51] (kind gift from Dr. Y. Takeuchi, University College London), we created a PERV-A 14/220* infectious clone by site-directed mutagenesis to introduce an F162S mutation in the Gag protein's second L domain. This clone has a 3.5-fold higher infectious titer on 293 T cells (25).

To generate GFP-tagged PAR cDNAs, we first PCR-amplified the enhanced GFP (eGFP) cDNA using primers that introduce a 5' *Kpn*I and a 3'*Apa*I site, digested with the respective enzymes and cloned into pcDNA3.1(+)/Zeo (Invitrogen) to generate pcDNA3.1(+)/Zeo eGFP. HuPAR1 (GenBank NP 078807) and HuPAR2 (GenBank Q9NWF4) cDNAs were amplified using primers that introduce a 5' *Hind*III site and 3'*Kpn*I site. The HuPAR2 template contained two amino acid polymorphisms, T261 and M296. *Hind*III and *Kpn*I were used to clone the cDNAs into pcDNA3.1(+)/Zeo eGFP immediately upstream of the eGFP cDNA. These constructs are referred to as HuPAR1eGFP and HuPAR2eGFP. The c-myc tag was inserted into the pcDNA3.1(+)/Zeo HuPAR2 backbone by site-directed mutagenesis with the following primer pair: 5'-CCAGCTTTGGGCTGAATGGAACAAAAACTTATTTCTGAAGAA GATCTGATGGCAGCACCCACG 3' and 5'-CGTGGGTGCTGCCATCAGATCTTCT TCAGAAATAAGTTTTTGTTCCATTCAGCCCAAAGCTGG-3'. MuPAR regions were introduced into the c-myc HuPAR2 or HuPAR2eGFP backbone by site-directed mutagenesis based on a megaprimer strategy [52]. Primer sequences used to generate the megaprimers are shown in Additional file 1, Table S1. Site-directed mutagenesis was used to introduce point mutations into the HuPAR2eGFP backbone and primer sequences are shown in Additional file 1, Table S2.

To create chimeric cDNAs, HuPAR1(HuPAR2 1–169)eGFP and HuPAR1(HuPAR2 170–448), the unique restriction site, *Xho*I, common to both HuPAR1 and HuPAR2 cDNA (n.t. 507–512) was used. HuPAR1eGFP and HuPAR2eGFP were digested with *Hind*III/*Xho*I and *Xho*I/*Kpn*I. Fragments were excised from a 2% agarose gel and purified with the QIAquick Gel Extraction Kit (Qiagen). Vector and insert were ligated using the Rapid DNA Ligation Kit (Roche) to yield HuPAR1(HuPAR2 1–169)eGFP and HuPAR1(HuPAR2 170–448). HuPAR2 regions were introduced into the HuPAR1eGFP backbone by site-directed mutagenesis that required prior amplification of the HuPAR2 sequence to create a megaprimer with 5' and 3' homology to HuPAR1 nucleotide sequence based on [52]. Primer sequences used to create the megaprimers as well as traditional site-directed mutagenesis primers to create HuPAR1(HuPAR2 ECL4)eGFP and the three amino acid insertion, KEE a.a. 245–247, are shown in Additional file 1, Table S3. All constructs were verified by sequencing.

### Assay for receptor function

Two hundred thousand cells, either naïve SIRC or SIRC cells stably expressing PAR cDNA, were plated in a 6-well plate. Twenty-four hours later, cells were exposed for four hours at 37°C to 1.0 mL supernatant harvested from 293 T cells chronically infected with PERV-A 14/220* supplemented with 8 μg/mL polybrene. PERV-containing supernatant was then removed and cells were washed three times with 2.0 mL PBS and replaced with fresh media. Seventy-two hours later, cells were detached and genomic DNA was purified with the DNeasy Kit (Qiagen). 250 ng genomic DNA was used for PERV *pol *detection by TaqMan quantitative PCR based on [53] with the following modifications: 20 μl total reaction volume and the TaqMan FastUniversal PCR Master Mix (2×) (Applied Biosystems). Reactions were run on the 7900 HT Real Time PCR System (Applied Biosystems). SIRC background PERV *pol *copy numbers were subtracted from each sample. All cell lines in a given experiment were normalized to the average wild-type receptor function as determined by PERV *pol *copy number.

### PERV SU-IgG assay for receptor binding

PERV SU-IgG fusion proteins were expressed and purified and binding was performed according to methods previously described (24). Briefly, 1–3 × 10^6 ^target cells were detached using 0.5 M EDTA, washed with PBS and fixed in 3% paraformaldehyde for 15 minutes. Cells were washed with PBS and 5% BSA sequentially and resuspended in 0.2–0.4 ml of 5% BSA containing a total of 500 ng of PERV SU-IgG per 10^6 ^cells and incubated for 1 hour on ice. The cells were washed twice with cold PBS containing 2% BSA and then incubated for 30 minutes on ice with anti-rabbit IgG antibody conjugated to Phycoerythrin (1:50 dilution) (Jackson ImmunoResearch). The cells were then washed 4 times with cold PBS containing 2% BSA. To determine PERV SU-rIgG binding, 10,000–15,000 live cell events were measured for Mean Channel Fluorescence on a FACScan (BD PharMingen) and analyzed using FlowJo (Tree Star Inc.). In these assays the PE signal generated by the full length PERV-A SU-rIgG was the metric for envelope binding and the eGFP signal was used to normalize for receptor expression. We then expressed the results as positive when the increase in the normalized PE channel signal was greater than or equal to twice the receptor-negative SIRC controls.

### Determination of HuPAR1 and HuPAR2 mRNA expression

Multiple human tissues were tested for relative HuPAR1 and HuPAR2 mRNA expression. Human colon, testes, lung, ovary and brain total RNA was purchased (Stratagene). Human peripheral blood lymphocyte (PBL), heart, liver and kidney were obtained as anonymous samples of purified RNA from an on-going, Scripps IRB-approved clinical study. Total RNA from these tissues was purified by Trizol (Invitrogen) extraction. Bone marrow was obtained from Dr. Edward Ball (University of California, San Diego) and was extracted using the RNeasy kit (Qiagen). 1 μg total RNA was used for cDNA amplification with the iScript cDNA Synthesis Kit (BioRad). The equivalent of 25 ng input RNA was used for TaqMan qPCR determination of HuPAR1 and HuPAR2 copy number. Samples were tested in triplicate. HuPAR1 primers and probe used were 5'-GCATGCTGTGCCTCGAATGTCACT-3' (forward) and 5'-GACCCAGGAAGAATGACCGTAAG-3' (reverse); HuPAR1 probe, 5'-FAM TTCTTGAGCCACCTGCCACCTCGC BHQ-3'. Underlined nucleotides represent differences between HuPAR1 and HuPAR2 in this region. HuPAR2 primers and probe used were 5'-GCCTGTTGTACCTCTAATGTCACT-3' (forward) and 5'-GACCCAGGAAGAAAGACCGTAAG-3' (reverse); HuPAR2 probe, 5'-FAM TTCCTGAGCCACCTGCCACCTCCT BHQ-3'. Final reaction concentrations were 200 nM probe and 300 nM primers in 20 μl total reaction volume with the TaqMan FastUniversal PCR Master Mix (2×) (Applied Biosystems). A ten-fold dilution series (10^1^-10^6^) of HuPAR1eGFP and HuPAR2eGFP plasmid DNA was used to create two standard curves. Comparisons of HuPAR1 and HuPAR2 copy numbers in different tissues are expressed relative to these standard curves. The average fold difference is expressed as an average of three patient samples (PBL, heart, liver, kidney and bone marrow) or the average of triplicates of single patient samples available commercially (colon, testes, lung, ovary and brain). Specificity of the primer/probe sets were as follows: a) HuPAR2 primer/probe set yielded <10 copies in a sample of 10^7 ^HuPAR1 copies and, b) the HuPAR1 primer/probe set yielded <10 copies in a sample of 10^4 ^HuPAR2 copies. Receptor-specific cDNA copy numbers detected in the all tissue compartments tested were below these thresholds.

## Results

### HuPAR2 exhibits greater function for PERV-A 14/220* infection than HuPAR1

**Figure 1:**
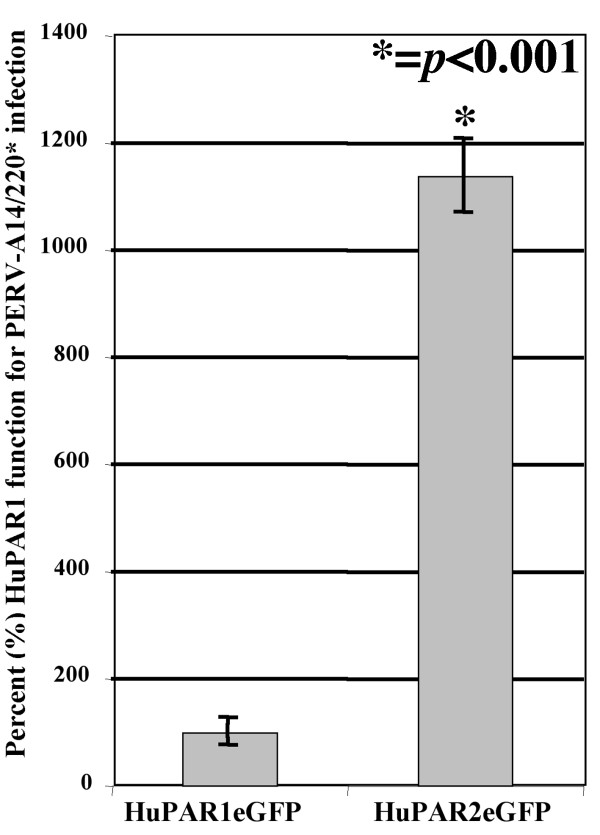
HuPAR1 and HuPAR2 function for PERV-A 14/220* infection. HuPAR1 and HuPAR2 C-terminally tagged eGFP constructs were expressed in non-permissive SIRC cells. Stable lines were sorted by eGFP expression to yield cell populations with similar receptor expression levels. PERV pol copy number in 250 ng genomic DNA of infected SIRC/receptor-expressing cell lines was determined to assess receptor function. HuPAR2 PERV pol copy number was normalized by HuPAR1 PERV pol copy number in each experiment and expressed as percent of HuPAR1 function. The average function determined by three individual infection experiments with three replicates each is shown with standard errors. HuPAR2 is 11-fold more functional than HuPAR1 (p < 0.001).

Full-length HuPAR1 and HuPAR2 with C-terminal eGFP tags were stably expressed in the non-permissive cell line, SIRC. C-terminal eGFP tags were used to sort homogenous cell populations with similar receptor expression levels. SIRC cells expressing either HuPAR1eGFP or HuPAR2eGFP were infected with supernatants from a stable producer line, PERV-A 14/220*. Seventy-two hours after infection, genomic DNA from the infected HuPAR1eGFP and HuPAR2eGFP SIRC cell lines was isolated. PERV *pol *copy numbers present in 250 ng of genomic DNA were determined by qPCR. HuPAR2eGFP PERV *pol *copy numbers were normalized by HuPAR1eGFP PERV *pol *copy numbers in each individual experiment (*n *= 3 with 3 replicates in each) and are expressed as percent of HuPAR1 function for PERV-A 14/220* infection (Figure 1). HuPAR2eGFP is 11-fold more functional for PERV-A 14/220* infection than HuPAR1eGFP (*p *< 0.001). However, we are not trying to over-emphasize the exact 11-fold number for this functional difference but rather that there is a consistent and significant difference in the functionality of HuPAR2 (from 5-fold to 15-fold in individual experiments) in every experiment performed.

### Increased HuPAR2 function is not due to increased PERV-A envelope binding

**Figure 2:**
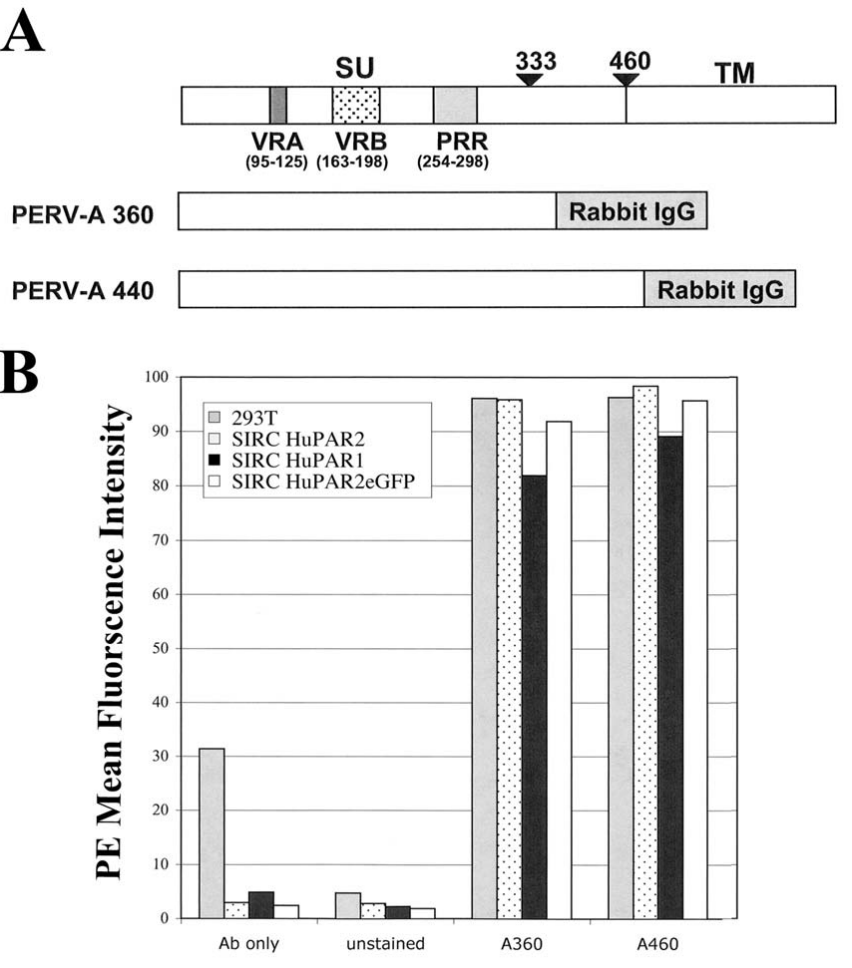
**In vitro PERV SU-IgG binding by HuPAR1 and HuPAR2**. (A) shows the SU constructs of either minimally-required (360 a.a.) or full-length (440 a.a.) PERV-A envelopes. All SU-IgG constructs contain Variable Region A (VRA), Variable Region B (VRB) and the Proline Rich Region (PRR). Binding of the soluble SU-IgG constructed is detected by a PE-conjugated secondary antibody that recognizes Rabbit IgG. (B) shows the Mean Fluorescence Intensity (MFI) detected by FACS and is representative of duplicate experiments. The 293 T cell line (gray bars) is a positive control for PERV-A binding. SIRC HuPAR2 (dotted bars) is a control for interference of the eGFP epitope tag in PERV-A binding. Both HuPAR1eGFP (black bars) and HuPAR2eGFP (white bars) bind PERV-A 360 and PERV-A 440 similar to the levels of 293 T and SIRC HuPAR2. Therefore, the difference between HuPAR1 and HuPAR2 in PERV-A 14/220* infection is not due to any difference in envelope binding.

To determine whether the average 11-fold increase in HuPAR2 function relative to HuPAR1, was due to increased binding of the PERV-A envelope protein, we measured the PERV SU rabbit-IgG (rIgG) binding. We recently reported that the regions of PERV-A envelope required for HuPAR recognition are Varible Region A (a.a. 95–125), Variable Region B (a.a. 163–198) and the Proline Rich Region (a.a. 254–298) [54]. Sorted SIRC cell lines expressing either HuPAR1eGFP or HuPAR2eGFP at equivalent levels were probed with various concentrations of various constructs of PERV SU-rIgG, followed by an anti-rabbit IgG PE-conjugated secondary antibody. Full-length SU, PERV-A 460, and truncated but functional SU, PERV-A 360, were used (Figure 2A). FACS was used to determine the Mean Fluorescence Intensity (MFI) of SU-IgG binding (Figure 2B). HuPAR1eGFP and HuPAR2eGFP display similar MFIs for both full-length and minimally required PERV-A SU-rIgG fusions. The PERV-A binding levels observed for HuPAR1eGFP and HuPAR2eGFP are similar to 293 T, which serves as a positive control for PERV-A SU-rIgG binding. These studies were always done at previously determined and optimal binding concentrations of ligand for this assay (24). PERV-A SU binding to SIRC/HuPAR2 and SIRC/HuPAR2eGFP was equivalent indicating that the receptor's C-terminal eGFP tag does not interfere with envelope binding. These results demonstrate that the increased viral entry function of HuPAR2 for PERV-A 14/220* infection is not due to an increase in virus binding.

### N-terminal 135 amino acids of HuPAR2 determine the functionality of the receptor

**Figure 3:**
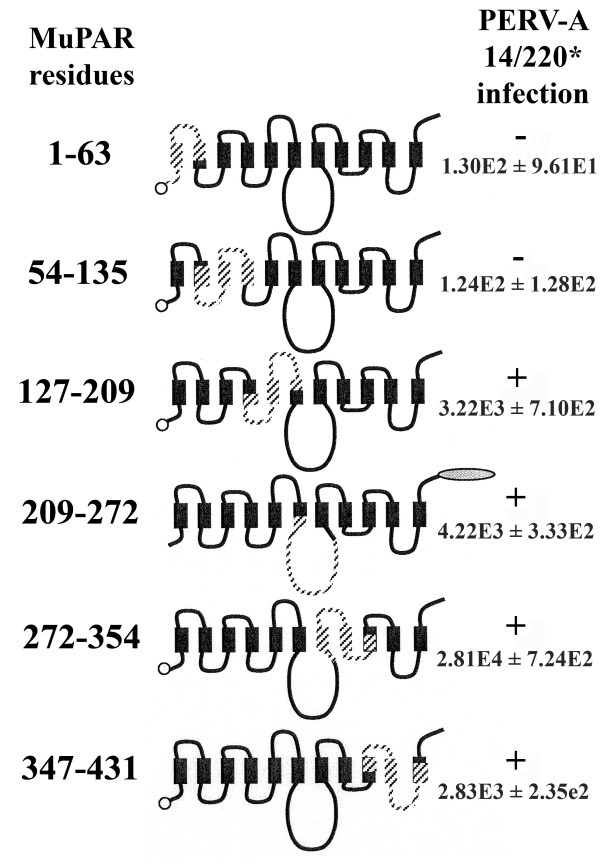
MuPAR and HuPAR2 chimeras reveal regions required for PERV-A 14/220* infection. MuPAR is not permissive for PERV-A binding and entry, while HuPAR2 is permissive for both. Chimeras were constructed by swapping regions of HuPAR2 (solid black) with the corresponding residues of MuPAR (hatched black). Constructs were tagged with either an N-terminal c-myc tag (open circle) or a C-terminal eGFP tag (gray oval), as a way to monitor expression. Chimeras were expressed in non-permissive SIRC cells and tested for PERV-A infection. Levels of infection were determined by PERV *pol *qPCR of 250 ng genomic DNA and compared to wild-type HuPAR2 and MuPAR. (-/+) indicates the status of PERV-A infection. The average PERV *pol *copy numbers and standard deviations (*n *= 3) are shown for each. These chimeras revealed that the N-terminal 135 amino acids are critical for PERV-A 14/220* infection.

Since HuPAR2 mediates PERV-A entry more efficiently than HuPAR1, the molecular determinants required for infection were mapped using chimeras of the permissive HuPAR2 and nonpermissive MuPAR. An N-terminal c-myc or a C-terminal eGFP epitope tag was used to monitor chimera expression levels. Regions of HuPAR2 were swapped with the homologous regions in MuPAR by mega-primer PCR mutagenesis. Six HuPAR2/MuPAR chimeras were constructed to scan the entire 448 amino acids of HuPAR2 (Figure 3). Tagged HuPAR2/MuPAR chimeras were expressed in SIRC cells and then assessed for PERV-A 14/220* infection levels by qPCR of PERV pol from genomic DNA. The first two HuPAR2/MuPAR chimeras, 1–63 and 54–135, were non-functional for PERV-A 14/220* infection. Thus, the N-terminal 135 amino acids are critical for PERV-A infection.

### Six structural regions in HuPAR2 impact PERV-A infection but only one alters PERV-A binding

**Figure 4:**
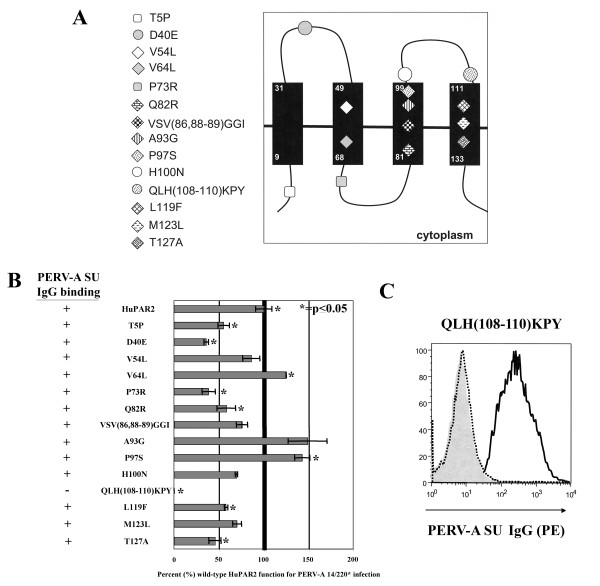
**Single residue and mini-region mapping of the eighteen amino acid differences in the critical N-terminal region of HuPAR2 for binding and infection**. (A) shows the location of the residue differences in HuPAR2 based on the current topology model. Mutations were introduced in the HuPAR2eGFP fusion protein and were expressed in non-permissive SIRC cells. Stably selected and eGFP sorted SIRC/HuPAR2 populations were assayed for PERV-A binding and infection by a FACS-based PERV-A SU IgG binding assay and a PERV *pol *qPCR-based infection assay. PERV *pol *copy numbers were normalized to wild-type HuPAR2 and expressed as percent (%) of wild-type (WT) HuPAR2 function. (B) shows the results from both the binding and infection assays (average of three replicates). Eight mutations significantly decreased HuPAR2 function for PERV-A infection (*p *≥ 0.05). Only one mutation, QLH(108–110)KPY, completely prevented PERV-A binding. (C) shows the FACS histogram from the binding assay. The PE fluorescence shift seen for wild-type HuPAR2 (solid black line) is not seen for QLH(108–110)KPY (dotted black line), which is identical to the SIRC cells not expressing a receptor (solid gray graph).

Within the critical N-terminal 135 amino acids, there are eighteen single amino acid differences between HuPAR2 and MuPAR. Figure 4A shows these amino acid differences and their predicted locations in HuPAR2. Each residue was tested individually or in clusters of three (i.e. mini-regions). Seventy-two hours after infection, genomic DNA was purified and PERV *pol *copy number in 250 ng was determined by qPCR. Results were normalized to that of wild-type HuPAR2eGFP and expressed as percent function for PERV-A 14/220* infection (Figure 4B). The same cell lines were used to assess PERV-A SU binding.

Results of the infection experiments revealed that seven mutations significantly decreased (*p *< 0.05) HuPAR2 function, expressed here as a percent of wild-type function: T5P (55%), D40E (36%), P73R (39%), Q82R (58%), QLH(108–110)KPY (0%), L119F (58%) and T127A (46%). Proper membrane orientation of these receptor mutants was verified by confirming that the C-terminal eGFP tag was extracellular (data not shown). With the single exception of QLH(108–110)KPY, the functional reductions were not due to a lack of PERV-A SU rIgG binding. Figure 4C shows the FACS analysis plot for the full length PERV-A SU-IgG binding assay of the QLH(108–110)KPY mutation (dotted line), the SIRC cell control (solid grey) and the binding wild-type receptor (solid black line). It is clear that the QLH(108–110)KPY mutation completely abolished PERV-A SU binding. The lack of both binding and infection of the QLH(108–110) mutation agrees with the previous report identifying L109 in the second extracellular loop as critical for mediating PERV-A entry [45]. Here we identify six additional residues that are also important in HuPAR2 function as a viral receptor.

### Fine mapping QLH(108–110 for PERV-A binding and infection

**Figure 5:**
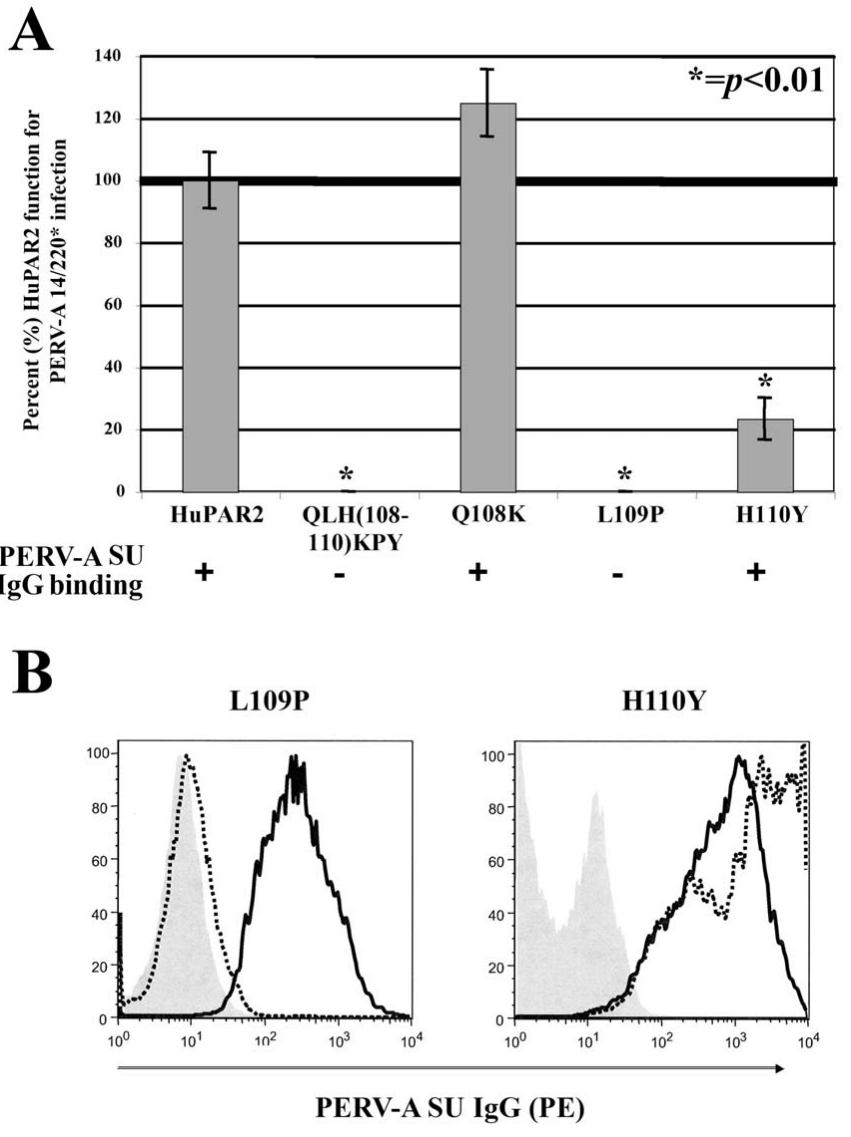
Contribution of QLH(108–110) to HuPAR2 function for PERV-A 14/220* infection at the single residue level. The individual requirement of each residue of the QLH(108–110) region to PERV-A binding and infection was determined. (A) shows both the percent (%) of wild-type (WT) HuPAR2 function and full-length PERV-A SU binding for QLH(108–110)KPY, Q108K, L109P and H110Y. The L109P mutant does not bind PERV-A SU. H110Y results in a significant decrease (*p *< 0.01) of HuPAR2 function for infection, but does not affect PERV-A SU binding. (B) shows the FACS histogram from the binding assay for both L109P and H110Y. The negative controls (naïve SIRC cells; gray shading) and L109P (dotted black line) shown in the first plot, indicate no binding of PERV-A SU IgG compared to HuPAR2eGFP (solid black line). In the second plot, H110Y (dotted black line) and the positive control, HuPAR2eGFP (solid black line) show equivalent SU IgG binding. Therefore, L109 is the only residue within the QLH mini-region that determines HuPAR2 binding.

Figure 5A shows the individual effects of Q108K, L109P and H110Y on HuPAR2 PERV-A binding and infection. Q108K does not affect PERV-A SU binding or HuPAR2 function for PERV-A infection. As previously reported [45], L109P completely abolished HuPAR2 function for PERV-A infection (*p *< 0.01) and abrogates envelope binding as shown in Figure 5B. In contrast, H110Y, which was not individually tested previously, significantly decreased HuPAR2 function for PERV-A infection by 77% relative to wild-type receptor. However, the decrease in infection for H110Y was not due to a lack of envelope binding (Figure 5B). Therefore, H110Y represents a functional determinant impacting a post-binding step.

We determined infectious titers using a beta-galactosidase pseudotyped PERV-A to confirm our qPCR assay with a second independent method. Titers are expressed as Blue Forming Units (BFU) per milliliter with the Standard Error (SE) averaged from two independent experiments performed in duplicate. The data in Table 1 confirms that QLH(108–110)KPY and L109P results in a complete loss of receptor function for infection and H110Y results in a 55% decrease in infection compared to wild-type (p < 0.0003).

**Table 1:** PERV-A lacZ pseudotype infectious titers of HuPAR2 constructs QLH(108–110)KPY, L109P and H110Y.

**HuPAR2eGFP construct**	**Average BFU^a^/mL ± SE^b^**	**Percent (%) HuPAR2 function**	***p *value compared to wild-type HuPAR2**
Wild-type	2.86 × 10^4 ^± 1.89 × 10^3^	100	
QLH(108–110)KPY	0 ± 0	0	*p *< 0.0003
L109P	0 ± 0	0	*p *< 0.0003
H110Y	1.28 × 10^4 ^± 1.58 × 10^3^	45	*p *< 0.0003

^a ^= Blue Forming Units

^b ^= Standard Error

### Mapping the region of HuPAR2 associated with increased PERV-A entry function compared to HuPAR1

**Figure 6:**
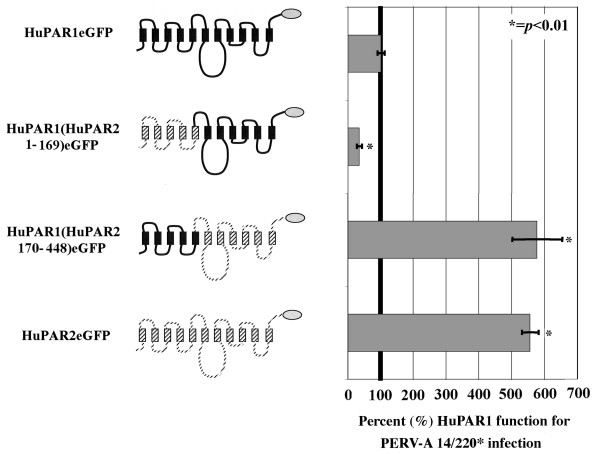
**HuPAR1 and HuPAR2 chimeras reveal that the C-terminal two-thirds of HuPAR2 is responsible for the increased functionality compared to HuPAR1**. eGFP-tagged chimeras (gray oval) were constructed between HuPAR1 (solid black) and HuPAR2 (dashed black). PERV-A 14/220* infection levels were determined for SIRC cells stably expressing each of the chimeric constructs. For purposes of comparison, we arbitrarily set the function of HuPAR1eGFP to 100%. The results indicate that HuPAR2 residues 170–448 contain the sequences responsible for the increased function for PERV-A infection.

While we showed above that there is no difference in envelope binding between HuPAR1 and HuPAR2, the expression of HuPAR2 in the non-permissive SIRC cells results in an average 11-fold increase in PERV infection (Figure 1). We constructed chimeras between HuPAR1 and HuPAR2 to determine the regions responsible for this difference. The first set of chimeras used a unique restriction site common to HuPAR1 and HuPAR2, *Xho*I, to create two chimeras roughly splitting the receptor in half as shown in Figure 6.

SIRC cell lines stably expressing either HuPAR1(HuPAR2 1–169)eGFP or HuPAR1(HuPAR2 170–448)eGFP were tested for infection. Figure 6 shows the results relative to HuPAR1 function set arbitrarily as 100%. HuPAR1(HuPAR2 1–169)eGFP exhibited a 64% decrease in function (*p *< 0.01) compared to HuPAR1eGFP demonstrating that the N-terminal region of HuPAR2, including all the determinants mapped above, is not responsible for the increased function observed. HuPAR1(HuPAR2 170–448)eGFP exhibited function equal to HuPAR2eGFP and significantly higher function than HuPAR1eGFP (*p *< 0.01). Therefore, the C-terminal half of the HuPAR2 molecule (a.a. 170–448) is responsible for the increased HuPAR2 function.

**Figure 7:**
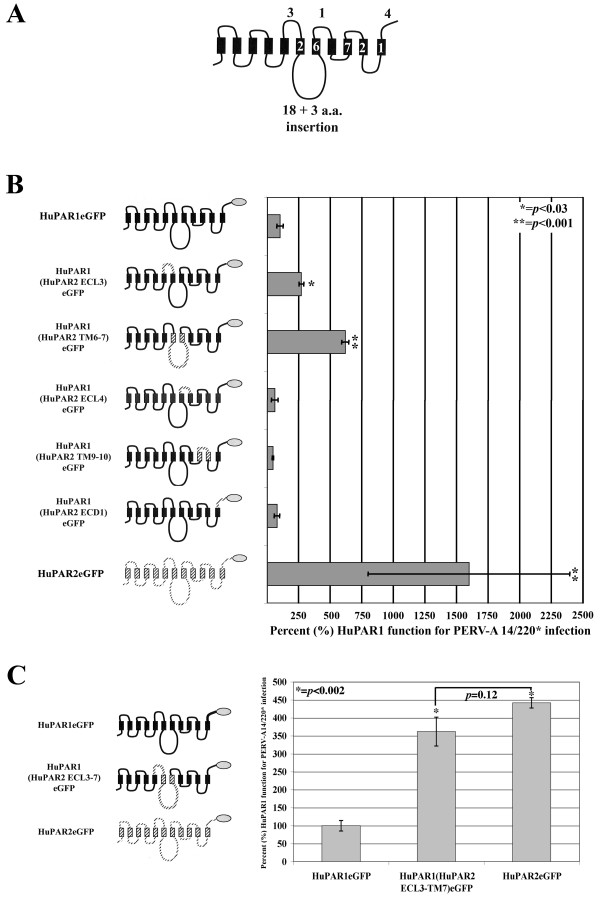
**Finer mapping of HuPAR2 residues 170–448 reveal that extracellular loop 3 (ECL3), and the region spanning transmembrane domain 6 and 7 (TM6–7), contribute to the increased function**. (A) shows the number of single amino acid differences for each structural region in the current topology model. (B) shows the eGFP-tagged chimeras (gray oval) constructed between HuPAR1 (solid) and HuPAR2 (dashed) used for mapping [transmembrane (TM), extracellular loop (ECL), intracellular loop (ICL), extracellular domain (ECD)]. Statistically significant increases were seen for ECL3 (p ≤ 0.03) and TM6–7 (p ≤ 0.001), implicating these regions as contributing to the increased functional efficiency of HuPAR2. (C) shows a statistically significant (p < 0.002) increase for infection, essentially to HuPAR2 wild-type levels, for the ECL3-TM7-containing chimera.

Of the 58 residues that distinguish HuPAR-1 and -2, 43 (74%) are found in C-terminal 338 residues (Figure 7A). We mapped this region with a series of HuPAR1/HuPAR2eGFP chimeras tested for infection (Figure 7B). HuPAR1(HuPAR2 TM9–10)eGFP, HuPAR1(HuPAR2 ECL4)eGFP and HuPAR1(HuPAR2 ECD1)eGFP were functionally equivalent to HuPAR1eGFP; therefore, the HuPAR2 regions in these chimeric receptors are not sufficient for the increased HuPAR2 function. In contrast, HuPAR1(HuPAR2 ECL3)eGFP and HuPAR1(HuPAR2 TM6–7)eGFP demonstrated statistically significant 2.6-fold (*p *< 0.03) and 6.2-fold increases (*p *< 0.001), respectively. However, neither of the HuPAR2 region chimeras, alone, was able to fully reconstitute HuPAR2 PERV-A infection levels in the HuPAR1 backbone.

Given the increases in HuPAR1 function by replacing either the third extracellular loop (ECL3) or the region containing transmembrane domain 6 (TM6), intracellular loop 3 (ICL3) and transmembrane domain 7 (TM7), we determined if combining these regions would produce wild-type levels of HuPAR2 function. Figure 7C demonstrates that expression of the HuPAR2 ECL3-TM6-ICL3-TM7 in the HuPAR1 backbone does indeed function as well as full length HuPAR2.

**Figure 8:**
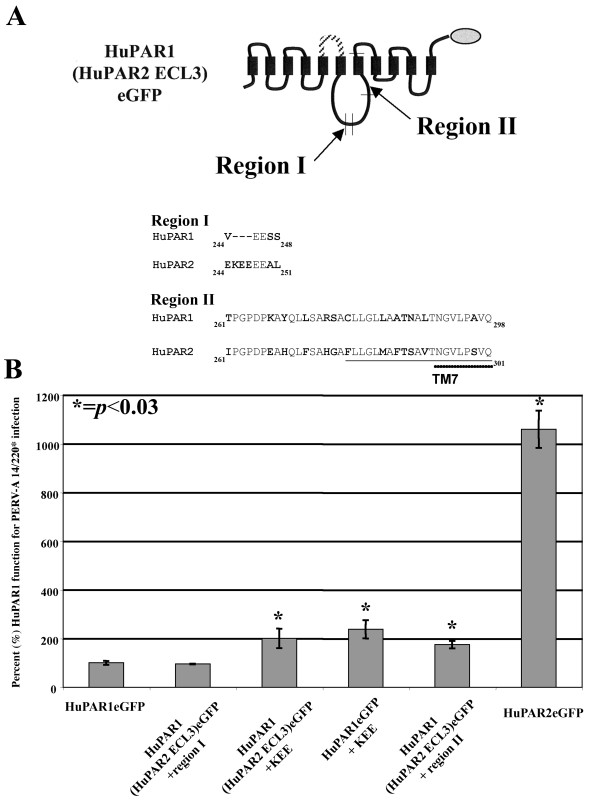
**Mapping the contribution of the third intracellular loop of HuPAR2 for PERV-A infection**. The third intracellular loop (ICL3) contains eighteen single amino acid differences, between HuPAR1 and HuPAR2, and a three amino acid insertion in HuPAR2. In order to determine what sequences in HuPAR2 ICL3 are required in addition to the third extracellular loop (ECL3) of HuPAR2, two HuPAR2 regions, I and II, were swapped. (A) shows the location of Regions I and II and the respective sequence differences (in bold) between HuPAR1 and HuPAR2. (B) shows chimera function for PERV-A 14/220* infection. Region II in the HuPAR1(HuPAR2 ECL3)eGFP backbone results in a statistically significant (p ≤ 0.03) increase, though neither Region confers full HuPAR2 function.

Comparison of amino acid residues of HuPAR-1 and -2 reveals that the region encompassing, ICL3-TM7 (Figure 7A) contains the most variation (24 differences plus a 3 amino acid insertion). Thus, we divided ICL3 and TM7 into Region I and Region II shown in Figure 8A and created chimeras using the HuPAR1 backbone containing the ECL3 of HuPAR2. We also created a chimera with the three amino acid insertion, KEE. Figure 8B shows that substitution of Region I, Region II or the KEE insertion into the HuPAR1(HuPAR2ECL3) chimera were not sufficient to restore PERV-A receptor function to the level of HuPAR2. Thus, the full sequence of HuPAR2 in this portion of the receptor's structure is required for the increased function.

### HuPAR1 mRNA expression is higher than HuPAR2 in multiple tissue compartments

Given two functional viral receptors, the infectious risk is dependent on the relative function of each receptor as well as their relative expression in target tissues. HuPAR1 and HuPAR2 expression in various human tissues was previously determined by Northern blot analysis using a pan-HuPAR probe [20]. We designed HuPAR1- and HuPAR2-specific primer and probe sets for RT-qPCR. The relative fold difference in mRNA expression for HuPAR1 and HuPAR2 transcripts in the ten tissue compartments tested is shown in Table 2. With the exception of testes, HuPAR1 mRNA expression is 2 to 50-fold higher than HuPAR2 in all tissues tested. Therefore, while expression of HuPAR2 in the non-permissive SIRC cell line demonstrates an average 11-fold advantage over HuPAR1 in mediating infection, the endogenous level of expression of HuPAR1 is significantly higher than this in many human tissues.

**Table 2:** Relative HuPAR1 and HuPAR2 mRNA expression levels in human tissues.

***Tissue***	***Relative fold difference (HuPAR1>HuPAR2)***
**Testes**	1
**Heart**	2
**Bone Marrow**	5
**Peripheral Blood Lymphocytes**	5
**Liver**	6
**Colon**	11
**Brain**	15
**Kidney**	18
**Ovary**	40
**Lung**	50

## Discussion

PERV-A, like other gammaretroviruses, exploits endogenous multi-membrane spanning cell-surface molecules to infect cells. Yet, PERV-A is one of a few gammaretroviruses that can enter human cells via either one of two receptors, HuPAR1 or HuPAR2. Moreover, PERV is an infectious risk to humans that would undergo cell, tissue or organ xenotransplantation from a porcine donor especially under intense immunosuppression. Therefore, the structure-function relationships of HuPAR1 and HuPAR2 are not only important to further a general understanding of gammaretroviral cell entry, but also, central to advancing a science-based risk-assessment for a productive PERV infection in human patients after xenotransplantation.

**Figure 9:**
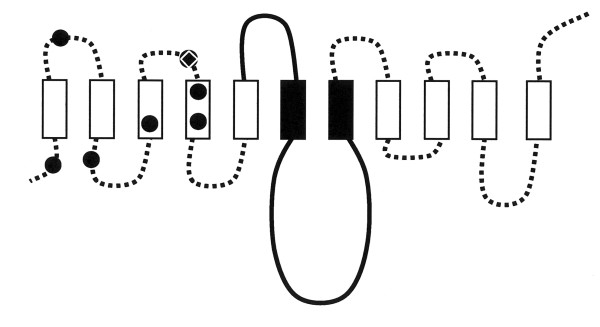
**Location of HuPAR2 residues and regions that determine HuPAR2 function identified to date**. HuPAR2 (no shading) has two regions involved in its function as a PERV-A receptor. The first N-terminal 135 amino acids contain the absolute requirements for viral receptor function. The role of L109 (open white diamond) as a determinant of PERV-A SU binding was confirmed. Residues T5, D40, P73, Q82, H110, L119 and T127 (black circles), identified by MuPAR/HuPAR2 chimeras, contribute to the efficiency of HuPAR2 function and are found in multiple structural elements. The second region (a.a. 152–285), identified by HuPAR1/HuPAR2 chimeras, determines the enhanced efficiency of HuPAR2 function for PERV-A infection. This region spans the third extracellular loop, sixth transmembrane domain, third intracellular loop and seventh transmembrane domain.

We identified two functionally distinct regions for PERV-A infection (Figure 9). The first region, identified by chimeras made with the non-permissive MuPAR ortholog, lies within the first N-terminal 135 amino acids. The N-terminal region contains both an absolute determinant of viral envelope binding (L109) as well as six additional residues that enhance the efficiency of PERV-A entry without any impact on envelope binding. The second region, identified by chimeras between the two permissive human PARs, is located in the middle of HuPAR2 (a.a. 152–285). This second region is responsible for the 11-fold increased function as compared to HuPAR1 and has no effect on PERV envelope binding.

The first region of HuPAR2 contains the envelope-binding determinant, L109, in the second extracellular loop. To date, the envelope binding regions of gammaretroviral receptors map to extracellular loops. For example, the first extracellular loop of Pit2 is important for binding of amphotropic MLV (A-MLV) [55]. Residues Y242 and E244 in the third extracellular loop of mCAT-1, the receptor for ecotropic MLV, are required for both binding and infection [56].

In contrast to the extracellular location of the envelope binding site, we found that multiple residues predicted to be within extracellular loops, intracellular loops or transmembrane domains influence HuPAR2 function for PERV-A infection. Within the N-terminal 135 amino acids, we identified seven single residues (T5, D40, P73, Q82, H110, L119 and T127) that determine the functional efficiency of HuPAR2 independent of affecting viral envelope binding. These residues are located in multiple structural regions (intracellular domain 1, extracellular loop 1, intracellular loop 1, and transmembrane regions 3 and 4). Based on the current literature, HuPAR2 is unique in having three types of structural features modulating receptor function. For example, GALV entry via Pit1 requires only an intracellular loop and a transmembrane region, denoted Regions A [41-43,57] and B [40] and within Region A, a single residue, D550, is involved [41,57-61]. Other gammaretroviral receptors, such as Pit2, FLVCR1 and X-receptor, require only extracellular loops for their function for A-MLV [55,62-64], FeLV-C [65] and X-MLV/P-MLV [38,66], respectively. The structural basis upon which residues determining the efficiency of HuPAR2 are found in intracellular, extracellular and transmembrane features is not readily apparent, as there are presently no crystallographic structures or empiric confirmation of predicted topological features for any of these receptors.

We show that HuPAR2 is on average 11-fold more functional than HuPAR1 for infection and that is not explained by any difference in viral envelope binding. The 11-fold functional superiority of HuPAR2 reflects the 5- to 15-fold range observed across the multiple experiments presented in this manuscript. This range in observed fold-differences between HuPAR2 and HuPAR1 is not due to differences in expression, as the initial expression levels selected for by eGFP did not change, but rather reflects the inherent biological variability in these viral infection testing strategies. The receptor determinants responsible for the significant increase in HuPAR2 function mapped to the second region that spans the third extracellular loop, sixth transmembrane domain, the third intracellular loop and the seventh transmembrane domain. Interestingly, the greatest degree of diversity between HuPAR1 and HuPAR2 sequence exists in this region. It was also clear that structural elements of the entire region, and not just a few discrete residues, are required for the enhanced PERV-A receptor function of HuPAR2.

While evidence for PERV-A infection in humans is lacking [1-5,7-12,67], long-term functional survival of a porcine cell or tissue xenotransplant has also not been achieved. Thus, it is still important to identify and characterize factors, such as receptor function and tissue expression, that could contribute to the infection risk in xenotransplantation while advances in the transplant science and immunosuppression are pursued to make clinical success possible. HuPAR1 and HuPAR2 expression in human tissues was originally determined by a pan-HuPAR Northern blot [20]. In this study, we determined relative expression levels for HuPAR1 and HuPAR2 using specific primer-probe sets. HuPAR1 mRNA was consistently expressed at 2 to 50-fold higher levels than HuPAR2 in the majority of the human tissues tested. Note that we are assuming at least a rough positive correlation between mRNA expression and cell-surface protein expression. We cannot easily test this assumption in human tissues as an antibody against HuPAR1 or HuPAR2 is not available. But we believe that the 11-fold higher activity of HuPAR2 for PERV infection is fairly balanced by a much higher tissue expression of HuPAR1 such that in clinical risk terms, both receptors represent significant portals for PERV entry.

## Conclusion

Our data show that multiple regions of HuPAR are required for optimal receptor function. We propose that the initial events of viral envelope binding are influenced by the L109 residue and several nearby residues, while subsequent events of viral fusion and entry impacting on receptor functionality are determined by additional residues within two separate regions of the receptor, the N-terminal 135 amino acids and the more complex structural features of a second region comprising ECL3 through TM7. Therefore, these results identify at least two distinct regions of receptor sequences necessary for the full process of viral binding and fusion that are all candidates for better understanding the structure/function determinants of this class of retroviral receptors and potentially developing novel anti-viral therapies.

## Competing interests

The authors declare that they have no competing interests.

## Authors' contributions

KTM carried out molecular biology to create receptor chimeras and mutations, selected stable cell lines, performed all experiments except receptor binding and BFU assays, participated in study design and drafted the manuscript. TA performed the receptor binding and BFU assays. DRS conceived of the study, was responsible for study design and refined the drafted manuscript. CAW contributed to study design and draft revision. All authors read and approved this manuscript.

## Supplementary Material

Additional file 1 **Supplemental methods. Tables showing HuPAR2/MuPAR megaprimers sequences, positive sense sequence of complementary primer pairs used to create HuPAR2 to MuPAR mutations and HuPAR1/HuPAR2 chimera primer sequences**. Click here for file

## References

[B1] Paradis K, Langford G, Long Z, Heneine W, Sandstrom P, Switzer WM, Chapman LE, Lockey C, Onions D, Otto E (1999). Search for cross-species transmission of porcine endogenous retrovirus in patients treated with living pig tissue. The XEN 111 Study Group. Science.

[B2] Patience C, Patton GS, Takeuchi Y, Weiss RA, McClure MO, Rydberg L, Breimer ME (1998). No evidence of pig DNA or retroviral infection in patients with short-term extracorporeal connection to pig kidneys. Lancet.

[B3] Irgang M, Sauer IM, Karlas A, Zeilinger K, Gerlach JC, Kurth R, Neuhaus P, Denner J (2003). Porcine endogenous retroviruses: no infection in patients treated with a bioreactor based on porcine liver cells. J Clin Virol.

[B4] Kuddus R, Patzer JF, Lopez R, Mazariegos GV, Meighen B, Kramer DJ, Rao AS (2002). Clinical and laboratory evaluation of the safety of a bioartificial liver assist device for potential transmission of porcine endogenous retrovirus. Transplantation.

[B5] Kuddus R, Patzer JF, Lopez R, Mazariegos GV, Meighen B, Kramer DJ, Fung JJ, Rao AS (2001). Valuation of transmission of porcine endogenous retrovirus into patients subjected to hemoperfusion using an extracorporeal bioartificial liver support system. Transplant Proc.

[B6] Levy MF, Argaw T, Wilson CA, Brooks J, Sandstrom P, Merks H, Logan J, Klintmalm G (2007). No evidence of PERV infection in healthcare workers exposed to transgenic porcine liver extracorporeal support. Xenotransplantation.

[B7] Levy MF, Crippin J, Sutton S, Netto G, McCormack J, Curiel T, Goldstein RM, Newman JT, Gonwa TA, Banchereau J, Diamond LE, Byrne G, Logan J, Klintmalm GB (2000). Liver allotransplantation after extracorporeal hepatic support with transgenic (hCD55/hCD59) porcine livers: clinical results and lack of pig-to-human transmission of the porcine endogenous retrovirus. Transplantation.

[B8] Pitkin Z, Mullon C (1999). Evidence of absence of porcine endogenous retrovirus (PERV) infection in patients treated with a bioartificial liver support system. Artif Organs.

[B9] Xu H, Sharma A, Okabe J, Cui C, Huang L, Wei YY, Wan H, Lei Y, Logan JS, Levy MF, Byrne GW (2003). Serologic analysis of anti-porcine endogenous retroviruses immune responses in humans after ex vivo transgenic pig liver perfusion. Asaio J.

[B10] Elliott RB, Escobar L, Garkavenko O, Croxson MC, Schroeder BA, McGregor M, Ferguson G, Beckman N, Ferguson S (2000). No evidence of infection with porcine endogenous retrovirus in recipients of encapsulated porcine islet xenografts. Cell Transplant.

[B11] Heneine W, Tibell A, Switzer WM, Sandstrom P, Rosales GV, Mathews A, Korsgren O, Chapman LE, Folks TM, Groth CG (1998). No evidence of infection with porcine endogenous retrovirus in recipients of porcine islet-cell xenografts. Lancet.

[B12] Di Nicuolo G, Kerkhove MP van de, Hoekstra R, Beld MG, Amoroso P, Battisti S, Starace M, di Florio E, Scuderi V, Scala S, Bracco A, Mancini A, Chamuleau RA, Calise F (2005). No evidence of in vitro and in vivo porcine endogenous retrovirus infection after plasmapheresis through the AMC-bioartificial liver. Xenotransplantation.

[B13] Dinsmore JH, Manhart C, Raineri R, Jacoby DB, Moore A (2000). No evidence for infection of human cells with porcine endogenous retrovirus (PERV) after exposure to porcine fetal neuronal cells. Transplantation.

[B14] Wilson CA, Wong S, VanBrocklin M, Federspiel MJ (2000). Extended analysis of the in vitro tropism of porcine endogenous retrovirus. J Virol.

[B15] Le Tissier P, Stoye JP, Takeuchi Y, Patience C, Weiss RA (1997). Two sets of human-tropic pig retrovirus. Nature.

[B16] Bosch S, Arnauld C, Jestin A (2000). Study of full-length porcine endogenous retrovirus genomes with envelope gene polymorphism in a specific-pathogen-free Large White swine herd. J Virol.

[B17] Czauderna F, Fischer N, Boller K, Kurth R, Tonjes RR (2000). Establishment and characterization of molecular clones of porcine endogenous retroviruses replicating on human cells. J Virol.

[B18] Akiyoshi DE, Denaro M, Zhu H, Greenstein JL, Banerjee P, Fishman JA (1998). Identification of a full-length cDNA for an endogenous retrovirus of miniature swine. J Virol.

[B19] Suzuka I, Shimizu N, Sekiguchi K, Hoshino H, Kodama M, Shimotohno K (1986). Molecular cloning of unintegrated closed circular DNA of porcine retrovirus. FEBS Lett.

[B20] Ericsson TA, Takeuchi Y, Templin C, Quinn G, Farhadian SF, Wood JC, Oldmixon BA, Suling KM, Ishii JK, Kitagawa Y, Miyazawa T, Salomon DR, Weiss RA, Patience C (2003). Identification of receptors for pig endogenous retrovirus. Proc Natl Acad Sci USA.

[B21] Oldmixon BA, Wood JC, Ericsson TA, Wilson CA, White-Scharf ME, Andersson G, Greenstein JL, Schuurman HJ, Patience C (2002). Porcine endogenous retrovirus transmission characteristics of an inbred herd of miniature swine. J Virol.

[B22] Albritton LM, Tseng L, Scadden D, Cunningham JM (1989). A putative murine ecotropic retrovirus receptor gene encodes a multiple membrane-spanning protein and confers susceptibility to virus infection. Cell.

[B23] Anderson MM, Lauring AS, Burns CC, Overbaugh J (2000). Identification of a cellular cofactor required for infection by feline leukemia virus. Science.

[B24] O'Hara B, Johann SV, Klinger HP, Blair DG, Rubinson H, Dunn KJ, Sass P, Vitek SM, Robins T (1990). Characterization of a human gene conferring sensitivity to infection by gibbon ape leukemia virus. Cell Growth Differ.

[B25] Miller DG, Edwards RH, Miller AD (1994). Cloning of the cellular receptor for amphotropic murine retroviruses reveals homology to that for gibbon ape leukemia virus. Proc Natl Acad Sci USA.

[B26] van Zeijl M, Johann SV, Closs E, Cunningham J, Eddy R, Shows TB, O'Hara B (1994). A human amphotropic retrovirus receptor is a second member of the gibbon ape leukemia virus receptor family. Proc Natl Acad Sci USA.

[B27] Rasko JE, Battini JL, Gottschalk RJ, Mazo I, Miller AD (1999). The RD114/simian type D retrovirus receptor is a neutral amino acid transporter. Proc Natl Acad Sci USA.

[B28] Tailor CS, Nouri A, Zhao Y, Takeuchi Y, Kabat D (1999). A sodium-dependent neutral-amino-acid transporter mediates infections of feline and baboon endogenous retroviruses and simian type D retroviruses. J Virol.

[B29] Battini JL, Rasko JE, Miller AD (1999). A human cell-surface receptor for xenotropic and polytropic murine leukemia viruses: possible role in G protein-coupled signal transduction. Proc Natl Acad Sci USA.

[B30] Quigley JG, Burns CC, Anderson MM, Lynch ED, Sabo KM, Overbaugh J, Abkowitz JL (2000). Cloning of the cellular receptor for feline leukemia virus subgroup C (FeLV-C), a retrovirus that induces red cell aplasia. Blood.

[B31] Tailor CS, Willett BJ, Kabat D (1999). A putative cell surface receptor for anemia-inducing feline leukemia virus subgroup C is a member of a transporter superfamily. J Virol.

[B32] Mendoza R, Anderson MM, Overbaugh J (2006). A putative thiamine transport protein is a receptor for feline leukemia virus subgroup A. J Virol.

[B33] Lauring AS, Anderson MM, Overbaugh J (2001). Specificity in receptor usage by T-cell-tropic feline leukemia viruses: implications for the in vivo tropism of immunodeficiency-inducing variants. J Virol.

[B34] Bae EH, Park SH, Jung YT (2006). Role of a third extracellular domain of an ecotropic receptor in Moloney murine leukemia virus infection. J Microbiol.

[B35] Eiden MV, Farrell K, Warsowe J, Mahan LC, Wilson CA (1993). Characterization of a naturally occurring ecotropic receptor that does not facilitate entry of all ecotropic murine retroviruses. J Virol.

[B36] Fischer N, Krach U, Niebert M, Tonjes RR (2003). Detection of porcine endogenous retrovirus (PERV) using highly specific antisera against Gag and Env. Virology.

[B37] Lundorf MD, Pedersen FS, O'Hara B, Pedersen L (1999). Amphotropic murine leukemia virus entry is determined by specific combinations of residues from receptor loops 2 and 4. J Virol.

[B38] Van Hoeven NS, Miller AD (2005). Use of different but overlapping determinants in a retrovirus receptor accounts for non-reciprocal interference between xenotropic and polytropic murine leukemia viruses. Retrovirology.

[B39] Marin M, Lavillette D, Kelly SM, Kabat D (2003). N-linked glycosylation and sequence changes in a critical negative control region of the ASCT1 and ASCT2 neutral amino acid transporters determine their retroviral receptor functions. J Virol.

[B40] Farrell KB, Russ JL, Murthy RK, Eiden MV (2002). Reassessing the role of region A in Pit1-mediated viral entry. J Virol.

[B41] Johann SV, van Zeijl M, Cekleniak J, O'Hara B (1993). Definition of a domain of GLVR1 which is necessary for infection by gibbon ape leukemia virus and which is highly polymorphic between species. J Virol.

[B42] Miller DG, Miller AD (1994). A family of retroviruses that utilize related phosphate transporters for cell entry. J Virol.

[B43] Pedersen L, Johann SV, van Zeijl M, Pedersen FS, O'Hara B (1995). Chimeras of receptors for gibbon ape leukemia virus/feline leukemia virus B and amphotropic murine leukemia virus reveal different modes of receptor recognition by retrovirus. J Virol.

[B44] Blond JL, Lavillette D, Cheynet V, Bouton O, Oriol G, Chapel-Fernandes S, Mandrand B, Mallet F, Cosset FL (2000). An envelope glycoprotein of the human endogenous retrovirus HERV-W is expressed in the human placenta and fuses cells expressing the type D mammalian retrovirus receptor. J Virol.

[B45] Mattiuzzo G, Matouskova M, Takeuchi Y (2007). Differential resistance to cell entry by porcine endogenous retrovirus subgroup A in rodent species. Retrovirology.

[B46] Hofmann K, Stoffel W (1993). TMbase–a database of membrane spanning proteins segments. Biol Chem Hoppe-Seyler.

[B47] Zhou H, Zhou Y (2003). Predicting the topology of transmembrane helical proteins using mean burial propensity and a hidden-Markov-model-based method. Protein Sci.

[B48] Tailor CS, Lavillette D, Marin M, Kabat D (2003). Cell surface receptors for gammaretroviruses. Curr Top Microbiol Immunol.

[B49] Overbaugh J, Miller AD, Eiden MV (2001). Receptors and entry cofactors for retroviruses include single and multiple transmembrane-spanning proteins as well as newly described glycophosphatidylinositol-anchored and secreted proteins. Microbiol Mol Biol Rev.

[B50] Andriamampandry C, Taleb O, Kemmel V, Humbert JP, Aunis D, Maitre M (2007). Cloning and functional characterization of a gamma-hydroxybutyrate receptor identified in the human brain. Faseb J.

[B51] Harrison I, Takeuchi Y, Bartosch B, Stoye JP (2004). Determinants of high titer in recombinant porcine endogenous retroviruses. J Virol.

[B52] Kirsch RD, Joly E (1998). An improved PCR-mutagenesis strategy for two-site mutagenesis or sequence swapping between related genes. Nucleic Acids Res.

[B53] Argaw T, Ritzhaupt A, Wilson CA (2002). Development of a real time quantitative PCR assay for detection of porcine endogenous retrovirus. J Virol Methods.

[B54] Gemeniano M, Mpanju O, Salomon DR, Eiden MV, Wilson CA (2006). The infectivity and host range of the ecotropic porcine endogenous retrovirus, PERV-C, is modulated by residues in the C-terminal region of its surface envelope protein. Virology.

[B55] Feldman SA, Farrell KB, Murthy RK, Russ JL, Eiden MV (2004). Identification of an extracellular domain within the human PiT2 receptor that is required for amphotropic murine leukemia virus binding. J Virol.

[B56] Albritton LM, Kim JW, Tseng L, Cunningham JM (1993). Envelope-binding domain in the cationic amino acid transporter determines the host range of ecotropic murine retroviruses. J Virol.

[B57] Tailor CS, Takeuchi Y, O'Hara B, Johann SV, Weiss RA, Collins MK (1993). Mutation of amino acids within the gibbon ape leukemia virus (GALV) receptor differentially affects feline leukemia virus subgroup B, simian sarcoma-associated virus, and GALV infections. J Virol.

[B58] Chaudry GJ, Farrell KB, Ting YT, Schmitz C, Lie SY, Petropoulos CJ, Eiden MV (1999). Gibbon ape leukemia virus receptor functions of type III phosphate transporters from CHOK1 cells are disrupted by two distinct mechanisms. J Virol.

[B59] Eiden MV, Farrell KB, Wilson CA (1996). Substitution of a single amino acid residue is sufficient to allow the human amphotropic murine leukemia virus receptor to also function as a gibbon ape leukemia virus receptor. J Virol.

[B60] Johann SV, Gibbons JJ, O'Hara B (1992). GLVR1, a receptor for gibbon ape leukemia virus, is homologous to a phosphate permease of Neurospora crassa and is expressed at high levels in the brain and thymus. J Virol.

[B61] Wilson CA, Farrell KB, Eiden MV (1994). Properties of a unique form of the murine amphotropic leukemia virus receptor expressed on hamster cells. J Virol.

[B62] Leverett BD, Farrell KB, Eiden MV, Wilson CA (1998). Entry of amphotropic murine leukemia virus is influenced by residues in the putative second extracellular domain of its receptor, Pit2. J Virol.

[B63] Lundorf MD, Pedersen FS, O'Hara B, Pedersen L (1998). Single amino acid insertion in loop 4 confers amphotropic murine leukemia virus receptor function upon murine Pit1. J Virol.

[B64] Tailor CS, Kabat D (1997). Variable regions A and B in the envelope glycoproteins of feline leukemia virus subgroup B and amphotropic murine leukemia virus interact with discrete receptor domains. J Virol.

[B65] Brown JK, Fung C, Tailor CS (2006). Comprehensive mapping of receptor-functioning domains in feline leukemia virus subgroup C receptor FLVCR1. J Virol.

[B66] Marin M, Tailor CS, Nouri A, Kozak SL, Kabat D (1999). Polymorphisms of the cell surface receptor control mouse susceptibilities to xenotropic and polytropic leukemia viruses. J Virol.

[B67] Dorling A, Riesbeck K, Warrens A, Lechler R (1997). Clinical xenotransplantation of solid organs. Lancet.

