# Supplementary material for: Identification of two distinct structural regions in a human porcine endogenous retrovirus receptor, HuPAR2, contributing to function for viral entry
Source: Retrovirology. 2009 Jan 14;6:3. doi: 10.1186/1742-4690-6-3 (PMC2630988; doi:10.1186/1742-4690-6-3)
Supplement: Additional file 1 — Supplemental methods. Tables showing HuPAR2/MuPAR megaprimers sequences, positive sense sequence of complementary primer pairs used to create HuPAR2 to MuPAR mutations and HuPAR1/HuPAR2 chimera primer sequences. [file 1742-4690-6-3-S1.doc]

**Supplemental methods**

Table S1. HuPAR2/MuPAR megaprimers sequences.

| **Primer name** | **Sequence** |
| --- | --- |
| MuPAR a.a. 1-63 (forward) | 5’-ATGGCAGCACCTCCGCTGGG-3’ |
| MuPAR a.a. 1-63 (reverse) | 5’-CAGCAGACCCAGGTTTCGCAGCG-3’ |
| MuPAR a.a. 54-153 (forward) | 5’- CAGCAGACCCAGGTTTCGCAGCG-3’ |
| MuPAR a.a. 54-153 (reverse) | 5’- GAAGGGCAGGAAAGTGACATTAGA-3’ |
| MuPAR a.a. 127-209 (forward) | 5’- TCTAATGTCACTTTCCTGCCCTTC-3’ |
| MuPAR a.a. 127-209 (reverse) | 5’- CCAGAAGGGCAGTCAACACCC-3’ |
| MuPAR a.a. 209-272 (forward) | 5’- GCCCTTCTGGGCACTTCAGCA-3’ |
| MuPAR a.a. 209-272 (reverse) | 5’- GAGAACAGCTGATGGGCCT-3’ |
| MuPAR a.a. 272-354 (forward) | 5’- AGGCCCATCAGCTGTTCTC-3’ |
| MuPAR a.a. 272-354 (reverse) | 5’- AAGAGCATGCCCAGCAGAGA-3’ |
| MuPAR a.a. 347-431 (forward) | 5’- TCTCTGCTGGGCATGCTCTT-3’ |
| MuPAR a.a. 347-431 (reverse) | 5’- AGATGCTGGTGGGAGGGAACA-3’ |

Table S2. Positive sense sequence of complementary primer pairs used to create HuPAR2 to MuPAR mutations

| **Primer** | **Sequence** |
| --- | --- |
| T5P | 5’- TGGCAGCACCCCCGCTGGGCCGT-3’ |
| D40E | 5’ CTGCCTGTGGTGGTAAAAGAGCTTCCAGAGGGTTGGAGC-3’ |
| V54L | 5’- CATACCTCTCTGTGCTTGTGGCGCTGGGA-3’ |
| V64L | 5’- CCTGGGTCTGCTGCTGGTGACCCTGTG-3’ |
| P73R | 5’-CTGTGGAGGCAGCTGGCCCGGGGCAAGGGCGAGCAGGTC-3’ |
| Q82R | 5’-CAGGTCCCCATCCGGGTGGTACAGGTG-3’ |
| VSV(86, 88-89)GGI | 5’- CCAGGTGGTACAGGGGCTGGGTATAGTGGGCACAGCC-3’ |
| A93G | 5’- AGTGGGCACAGGCCTGCTGGCCC-3’ |
| P97S | 5’- GCCCTGCTGGCCTCTCTGTGGCACC-3’ |
| H100N | 5’- GCCCCTCTGTGGAACCACGTGGCCC-3’ |
| QLH(108-110)KPY | 5’- GTGGCCCCAGTGGCAGGGAAGCCCTACTCTGTGGCCTTCCTAAC-3’ |
| Q108K | 5’- CCCAGTGGCAGGGAAGCTCCACTCTGT-3’ |
| L109P | 5’- TGGCAGGGCAGCCCCACTCTGTGGC-3’ |
| H110Y | 5’- GGCAGGGCAGCTCTACTCTGTGGCCTT-3’ |
| L119F | 5’- CCTAACTCTGGCCTTCGTGTTGGCAATGGC-3’ |
| M123L | 5’- CAACAGGCCAGTGCCAACACCAAGGCCAG-3’ |
| T127A | 5’- GTTGGCAATGGCCTGTTGTGCCTCTAATGTCACTT-3’ |

Table S3. HuPAR1/HuPAR2 chimera primer sequences.

| Primer | Sequence |
| --- | --- |
| HuPAR2 ECL3 (forward) | 5’- GTGCTGGCCCTAGTGCAGGTG-3’ |
| HuPAR2 ECL3 (reverse) | 5’- AGTGCCCAGAAGAAGGTGCTGGC-3’ |
| HuPAR2 TM6-7 (forward) | 5’- GACTGCCCTTCTGGTCACTTCA-3’ |
| HuPAR2 TM6-7 (reverse) | 5’- CAGGCCCAGCAGGAAGGC-3’ |
| HuPAR2 TM9-10 (forward) | 5’- CTGGCTGTGGTGCTGGGCAG-3’ |
| HuPAR2 TM9-10 (reverse) | 5’- CTGCCAGCAATGCCGGCC-3’ |
| HuPAR2 ECD1 (forward) | 5’- ATGTTCCCTCCCACCAGCATC-3’ |
| HuPAR2 ECD1 (reverse) | 5’- GCCGTCCAGCTCGACCAGGA-3’ |
| Region I (forward) | 5’- CCAGGAGCAGAGGAGGAA-3’ |
| Region I (reverse) | 5’-GGTGCCTGTTGCCTGGCT-3’ |
| Region II (forward) | 5’- CCATTGCAGGAGCCACCG-3’ |
| Region II (reverse) | 5’- CAAACAGGAAAAGCTCTGCAC-3’ |
| HuPAR2 ECL4 (forward) | 5’-CAATGGCGTGCTGCCTTCCGTGCAGAG-3’ |
| HuPAR2 ECL4 (reverse) | 5’- CTCTGCACGGAAGGCAGCACGCCATTG-3’ |
| KEE a.a. 245-247 (forward) | 5’- GCAGAGGAAGAGGTGAAGGAGGAAGAAGAGTCC-3’ |
| KEE a.a. 245-247 (reverse) | 5’- GGACTCTTCTTCCTCCTTCACCTCTTCCTCTGC-3’ |
